# Reply to: Towards solving the missing ice problem and the importance of rigorous model data comparisons

**DOI:** 10.1038/s41467-022-33954-x

**Published:** 2022-10-24

**Authors:** Evan J. Gowan, Xu Zhang, Sara Khosravi, Alessio Rovere, Paolo Stocchi, Anna L. C. Hughes, Richard Gyllencreutz, Jan Mangerud, John-Inge Svendsen, Gerrit Lohmann

**Affiliations:** 1 Department of Earth and Environmental Sciences, Kumamoto University, Kumamoto, Japan; 2 Alfred Wegener Institute, Helmholtz Center for Polar and Marine Research, Bremerhaven, Germany; 3 MARUM, University of Bremen, Bremen, Germany; 4 Key Laboratory of Western China’s Environmental Systems (Ministry of Education), College of Earth and Environmental Science, Centre for Pan Third Pole Environment (Pan-TPE), Langzhou University, Langzhou, China; 5 Alfred Wegener Institute, Helmholtz Center for Polar and Marine Research, Potsdam, Germany; 6 DAIS, Ca’ Foscari University of Venice, Venice, Italy; 7 NIOZ, Texel, Netherlands; 8 Department of Geography, University of Manchester, Manchester, UK; 9 Department of Geological Sciences, Stockholm University, Stockholm, Sweden; 10 Department of Earth Science, University of Bergen and Bjerknes Centre for Climate Research, Bergen, Norway

**Keywords:** Cryospheric science, Palaeoclimate, Geodynamics

**replying to** Y.Yokoyama et al. *Nature Communications* 10.1038/s41467-022-33952-z (2022)

Our recent ice sheet reconstruction, PaleoMIST 1.0, was created on the basis of using near-field (i.e., ice sheet proximal) geological constraints. This was done so that it would be independent of far-field relative sea level observations, that are subject to uncertainties in the global distribution of ice, and deep sea proxy based global mean sea level reconstructions, which have large uncertainties due to temperature and salinity effects. We do not disagree with the interpretation of the far-field data highlighted by Yokoyama et al., but emphasise that near-field constraints should be the starting point for reconstructing ice sheets.

We thank Yokoyama et al. for the opportunity to further discuss our ice sheet and paleotopography reconstruction, PaleoMIST 1.0^1^ and acknowledge their extensive work acquiring sea level proxy data.

Yokoyama et al. state: community efforts have led to better understanding of the GMSL (e.g., PALSEA). We agree, and this is why we provided a comparison of our modelled sea level against scrutinised paleo relative sea level proxies for over 150 regions^2^, primarily taken from databases assembled by the HOLSEA project^3^. We focused on including datasets that we used to reduce the misfit with modelled near-field relative sea level in North America^4,5^ and Europe^6–8^. We included a far-field dataset from southeastern Asia^9^ and selected locations in tropical regions based on a database of coral relative sea level proxies^10^, including Tahiti and the Huon Peninsula. This model-data comparison was used to justify the Earth model used in our reconstruction.

No standardised database exists for the LGM, so we entered data from a few well known far-field areas to test if the ice sheet volume in our reconstruction was reasonable. This was neither claimed nor meant to be a comprehensive review, and we unintentionally missed adding some data from the Bonaparte Gulf^11^. We do dispute the interpretation of Yokoyama et al. that relative sea level lowstand was between −120.6 and −124.5 m at the location of core GC5 in the Bonaparte Gulf. Here, we have included this data, along with several other far-field sites (Fig. 1).

**Fig. 1:**
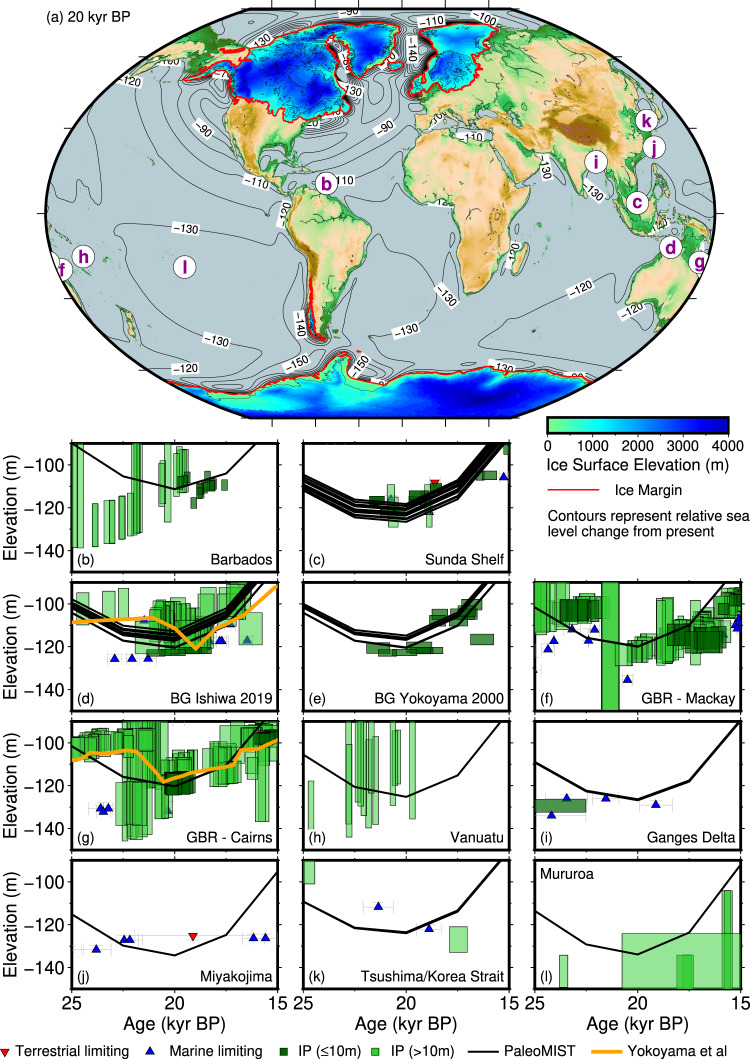
Last Glacial Maximum paleotopography reconstruction from PaleoMIST, and far-field relative sea level proxies. The minimum sea level in this model happens at 20 kyr BP (thousands of years before present). Since we calculate sea level at the location of each observation, in some locations there is a range of values if there is a regional gradient. The orange lines represent the sea level curves in the comment by Yokoyama et al. Index points (IP) have been separated based on whether the water depth range uncertainty is below or above 10 m. Error ranges represent 2-sigma uncertainties. **a** Paleotopography reconstruction. **b** Barbados^28,29^. **c** Sunda Shelf^30–32^. **d** Bonaparte Gulf (BG)^11,13^ using the interpretation from ref. 13. **e** Bonaparte Gulf (BG)^11^ using the interpretation from ref. 11. **f** Great Barrier Reef (GBR) near Mackay^12^. **g** GBR at Cairns^12^. **h** Vanuatu, using coral depth range from refs. 10,33,34. **i** Ganges Delta^35^. **j** Miyakojima^36^. **k** Tsushima/Korea Strait^37^. **l** Mururoa, using coral depth range from refs. 10,38. The figure is plotted using Generic Mapping Tools^27^.

For the Great Barrier Reef, when converting the data from ref. 12 to index points, we made an error by subtracting half of the water depth range estimate instead of adding. As a result, the index points plotted below the depth of the sample, instead of above. We apologise to Yokoyama et al. for this error. We do not dispute their interpretations. The corrected plot is shown in Fig. 1.

Originally, we conservatively set proxies with large uncertainties (i.e., >10 m)^10,13^ to be marine limiting (i.e., sea level was above the elevation of the indicator). Such large uncertainties reduce the utility of these data to precisely define paleo sea level. Here, we plot them as sea level indicators (index points), using different colours for data with vertical uncertainties below and above 10 m.

The model-data comparison shown in Fig. 1 demonstrates that the calculated relative sea level from our ice sheet reconstruction is consistent with many of the available proxies that constrain far-field LGM sea level to be between −100 and −130 m. Specific to this comment, the calculated minimum relative sea level with our preferred Earth model is −117 m at the location of core GC5 in the Bonaparte Gulf (Yokoyama et al.’s estimate is −120 to −123 m), and −120 m off the coast of Cairns (Yokoyama et al.’s estimate is −118 m). The discrepancy between our modelled sea level and the Bonaparte Gulf proxy can plausibly be explained by the lack of ocean thermal expansion effects, groundwater storage changes, and the absence of smaller ice caps and glaciers in our reconstruction, estimated to be 3–4 m of sea level equivalent at the LGM^14^.

Figure 2 shows relative sea level at a number of locations between 57 and 27 kyr BP (covering Marine Isotope Stage (MIS) 3). Some of the data support the deep sea *δ*^18^O records, while some support sea level that is 10 s of metres higher. For Papua New Guinea, we have plotted the data as interpreted in three different studies^10,15,16^. Our calculated relative sea level during MIS 3 is higher than estimates presented by ref. 16, but is consistent with the revised estimates from ref. 10, and ref. 17. For Tahiti, our modelled relative sea level is consistent with the estimate pointed out by Yokoyama et al. (although the estimate in ref. 18 was −67 to −101 m, not −65 to −75 m). This proxy is from the final part of MIS 3 when the ice sheets were advancing, and does not represent the MIS 3 highstand period.

**Fig. 2:**
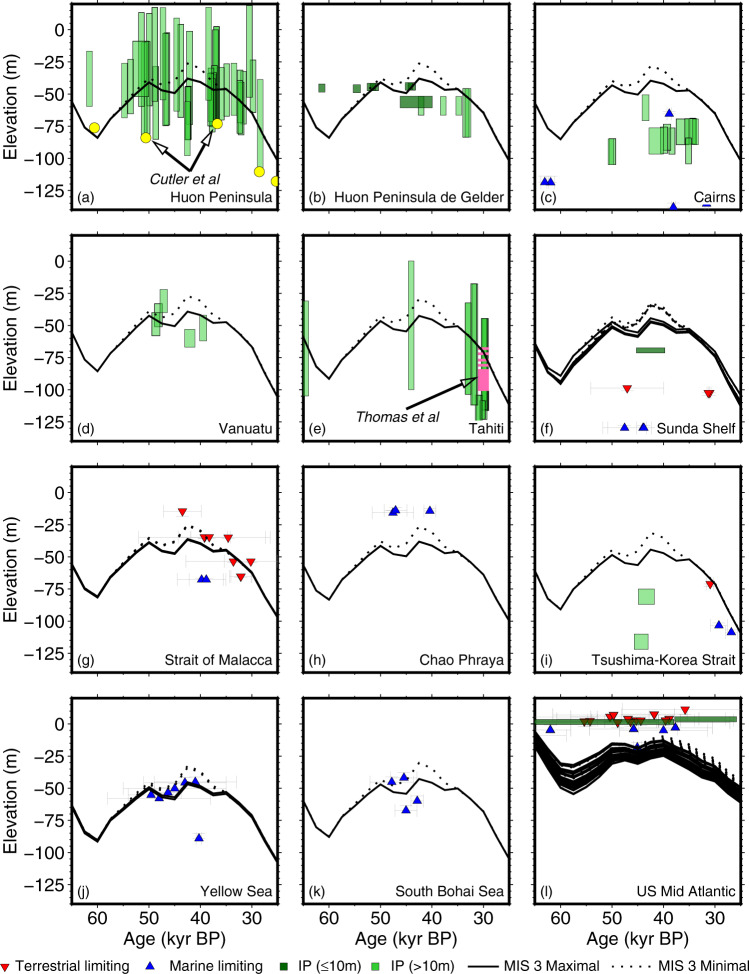
Relative sea level proxies from Marine Isotope Stage 3. This period is between 57–27 kyr BP (thousands of years before present). Index points (IP) have been separated based on whether the water depth range uncertainty is below or above 10 m. Error ranges represent 2-sigma uncertainties. The solid lines represent the PaleoMIST maximal scenario (Hudson Bay remains ice covered), and the dotted line is the minimal scenario (Hudson Bay is ice free for a period). **a** Huon Peninsula, Papua New Guinea, using coral depth range estimates from refs. 10,15,39,40. The yellow circles show the sea level estimates from ref. 15 based on terrace elevations. **b** Huon Peninsula, from marine terraces and revised uplift rates from ref. 16. **c** Cairns, Australia^12^. **d** Vanuatu^41^. **e** Tahiti, French Polynesia, using coral depth range from Hibbert et al., and including the MIS 3 depth range estimate from Thomas et al. in pink^10,17^. **f** Sunda Shelf, Southeast Asia^31,42^ (**g**) Strait of Malacca, Southeast Asia^9,43^. **h** Chao Phraya, Southeast Asia^9,44^. **i** Tsushima/Korea Strait, Eastern Asia^37^. **j** Yellow Sea, Eastern Asia^45–47^. **k** South Bohai Sea, Eastern Asia^47,48^. **l** Mid-Eastern United States^49–57^. The figure is plotted using Generic Mapping Tools^27^.

The geological constraints of limited ice sheet extent make it implausible for global average sea level to be −60 to −90 m during most of MIS 3^18^, even when accounting for two different hypotheses for Laurentide Ice Sheet configuration^19,20^. It is possible to increase the ice volume in our model by increasing the basal shear stress. We increased the maximal scenario values by 20%, but this only lowered sea level by 5.2 m. The core region of the Laurentide Ice Sheet was likely warm-bedded through the glacial cycle^21^, so it is unlikely that this could be invoked to significantly increase ice volume.

Our reconstruction was based only on near-field constraints. One reason for this was so that it would be independent of deep sea foraminifera *δ*^18^O records. *δ*^18^O_*foram*_ reflects changes in ambient (deep water) temperature as well as the oxygen isotopic composition of seawater, which itself is a function of global ice volume and water mass mixing^22–24^. A second reason is that sea level proxies prior to about 12 kyr BP are rare and subject to uncertainties due to tectonics and sediment loading, and the ~40 kyr limit of the radiocarbon method. The third reason is that the available LGM (and MIS 3) records are ambiguous as to where the water is distributed between the ice sheets^25^. There are significant differences in the Earth structure between ice sheets and locations where far-field relative sea level records exist^26^. Therefore, it is questionable if sea level calculated using spherically symmetric Earth structures (used by us and by Yokoyama et al.) can precisely represent far-field sea level. Finally, our models do not include non-ice sheet and GIA sources of water volume changes, which will lead to an inherent uncertainty on sea level of several metres^14^. This is why we used these proxies qualitatively to test our ice sheet reconstruction, rather than as an absolute constraint.

We consider our model as preliminary and we expect different results in future reconstructions with different assumptions on Earth model and ice sheet margin configuration. This is demonstrated by the calculated sea level lowstand at the Bonaparte Gulf and GBR sites (Fig. 1), which is similar to Yokoyama et al’s despite having a different ice sheet configuration. This is what led us to conclude there is no LGM “missing ice problem”, and that the solution to global ice volume at the LGM may be non-unique given the current constraints.

Ultimately, the solution to reducing the uncertainties on past sea level and ice sheet configuration is to collect new data. Yokoyama et al are providing a great service to the community with their efforts to do this. However, though far-field sea level proxies are a valuable resource to deduce global ice volume through time, they should not be used in exclusion of glacial-geological and near-field sea level observations, which we believe are the fundamental starting point for ice sheet reconstruction.

## Data Availability

Updated versions of the two reports comparing calculated sea level and sea level proxies at over 150 locations^2^, which includes a description of the evaluation methods, are available at 10.5281/zenodo.5647136. The scripts and paleo sea level proxy database used to create these reports are available at https://github.com/evangowan/paleo_sea_level. Source data are provided with this paper.

## Source data

source data
