# Supplementary figures and images for: Reply to: Towards solving the missing ice problem and the importance of rigorous model data comparisons
Source: Nat Commun. 2022 Oct 24;13:6264. doi: 10.1038/s41467-022-33954-x (PMC9592598; doi:10.1038/s41467-022-33954-x)

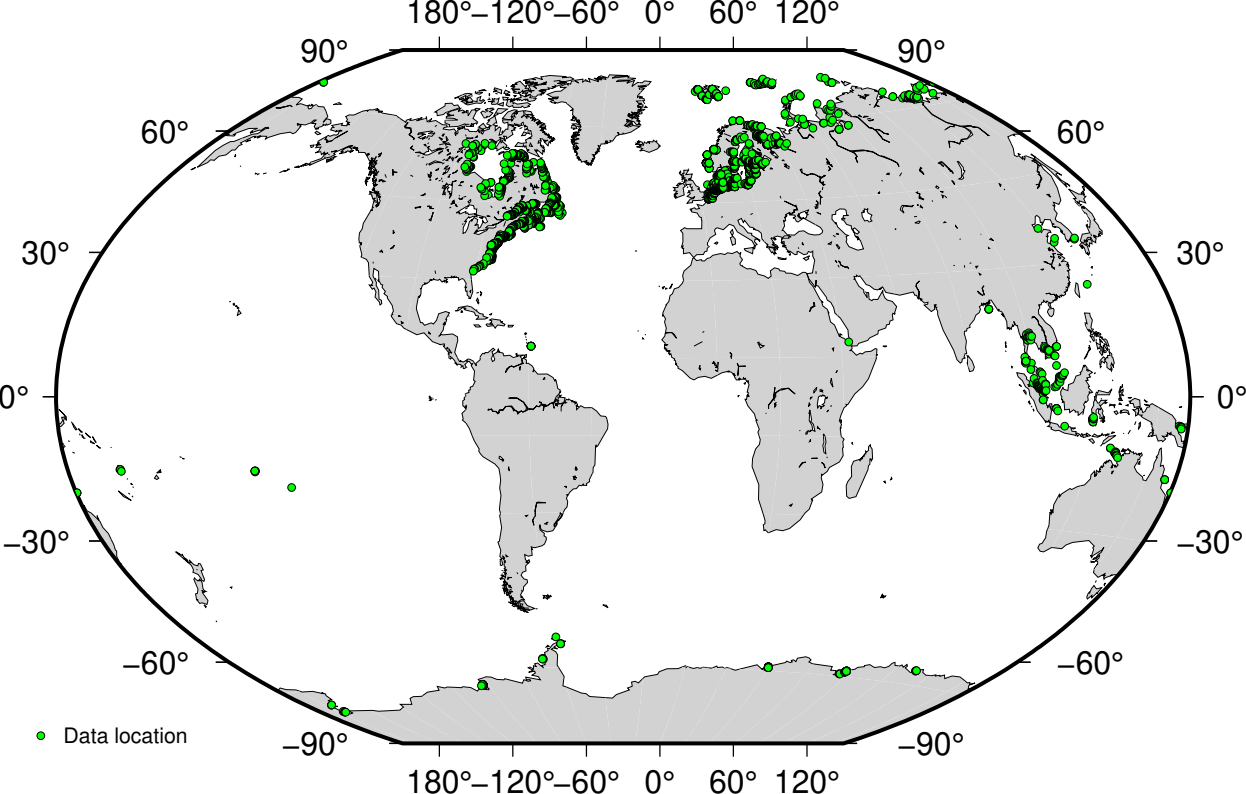

Supplement: Supplementary file 1 — source data [file 41467_2022_33954_MOESM1_ESM.zip › paleo_sea_level-1.3/GIS/data_map.pdf]
